# Percutaneous Thermal Ablation Therapy of Hepatocellular Carcinoma (HCC): Microwave Ablation (MWA) versus Laser-Induced Thermotherapy (LITT)

**DOI:** 10.3390/diagnostics12030564

**Published:** 2022-02-23

**Authors:** Hamzah Adwan, Thomas J. Vogl, Ümniye Balaban, Nour-Eldin Abdelrehim Nour-Eldin

**Affiliations:** 1 Department of Diagnostic and Interventional Radiology, University Hospital, Goethe University, Theodor-Stern-Kai 7, 60590 Frankfurt, Germany; adwan.hamza97@gmail.com (H.A.); mohammed.nour@kgu.de (N.-E.A.N.-E.); 2 Department of Biostatistics and Mathematical Modeling, University Hospital, Goethe University, Theodor-Stern-Kai 7, 60590 Frankfurt, Germany; balaban@med.uni-frankfurt.de; 3 Department of Diagnostic and Interventional Radiology, Cairo University Hospital, Cairo 12613, Egypt

**Keywords:** hepatocellular carcinoma, percutaneous thermal ablation, microwave ablation, laser-induced thermotherapy

## Abstract

The purpose of this study is to compare the efficacy and safety of microwave ablation (MWA) versus laser-induced thermotherapy (LITT) as a local treatment for hepatocellular carcinoma (HCC,) with regard to therapy response, survival rates, and complication rates as measurable outcomes. This retrospective study included 250 patients (52 females and 198 males; mean age: 66 ± 10 years) with 435 tumors that were treated by MWA and 53 patients (12 females and 41 males; mean age: 67.5 ± 8 years) with 75 tumors that were treated by LITT. Tumor response was evaluated using CEMRI (contrast-enhanced magnetic resonance imaging). Overall, 445 MWA sessions and 76 LITT sessions were performed. The rate of local tumor progression (LTP) and the rate of intrahepatic distant recurrence (IDR) were 6% (15/250) and 46% (115/250) in the MWA-group and 3.8% (2/53) and 64.2% (34/53) in the LITT-group, respectively. The 1-, 3-, and 5-year overall survival (OS) rates calculated from the date of diagnosis were 94.3%, 65.4%, and 49.1% in the MWA-group and 96.2%, 54.7%, and 30.2% in the LITT-group, respectively (*p*-value: 0.002). The 1-, 2-, and 3-year disease-free survival (DFS) rates were 45.9%, 30.6%, and 24.8% in the MWA-group and 54.7%, 30.2%, and 17% in the LITT-group, respectively (*p*-value: 0.719). Initial complete ablation rate was 97.7% (425/435) in the MWA-group and 98.7% (74/75) in the LITT-group (*p*-value > 0.99). The overall complication rate was 2.9% (13/445) in the MWA-group and 7.9% (6/76) in the LITT-group (*p*-value: 0.045). Based on the results, MWA and LITT thermal ablation techniques are well-tolerated, effective, and safe for the local treatment of HCC. However, MWA is recommended over LITT for the treatment of HCC, since the patients in the MWA-group had higher survival rates.

## 1. Introduction

Hepatocellular carcinoma (HCC) is the most common primary malignant tumor of the liver and the sixth most common cancer [1] that typically occurs in a cirrhotic liver [2]. The incidence of HCC is increasing worldwide, with the highest rates reported in Asia and Africa [3]. HCC is up to 8 times more often in men than women [1].

Liver transplantation (LT) is most suitable for patients within the Milan criteria and surgical resection for patients with solitary HCC and adequate liver function [4]. Local ablative treatments, such as microwave ablation (MWA), radiofrequency ablation (RFA), and laser-induced thermotherapy (LITT), can be performed in the case of unresectable or early-stage HCC [1]. Local ablation therapies could be curatively applied in patients with HCC [5].

Transarterial chemoembolization (TACE) can be carried out as a combined treatment for HCC before local ablative treatments to downsize the tumor, as bridging to LT, or palliatively by high tumor burden [6]. In patients with advanced tumor stage, non-invasive therapies, such as 3-Dimensional Conformal Radiation Therapy (3D-CRT), can also be an appropriate treatment option for HCC [1].

While treatments using MWA and RFA of HCC have been investigated in many studies worldwide, the effects of LITT of HCC has been less studied [7]. The number of studies which evaluate LITT of HCC and compared to MWA are too low to thoroughly evaluate the efficacy and safety of HCC treatments. The aim of this study is to compare MWA with LITT of HCC according to survival rates, tumor response, and complications.

## 2. Materials and Methods

### 2.1. Ethical Statement

This study was approved by the ethics committee of our university hospital.

### 2.2. Patient and Tumor Characteristics

In this retrospective study, we enrolled a total of 303 patients with histologically diagnosed HCC: 250 patients (52 females and 198 males; mean age: 66 ± 10 years) with 435 tumors that were treated by 445 MWA sessions and 53 patients (12 females and 41 males; mean age: 67.5 ± 8 years) with 75 tumors that were treated by 76 LITT sessions, with the intention of local tumor control. The enrolled patients were similar without significant differences regrading gender, age, size, or number of tumors between both groups. We included patients that were in the early or intermediate tumor stages with (1) HCC lesions with a maximum axial diameter of 5 cm, (2) a maximum number of 5 lesions, and (3) adequate coagulation (international normalized ratio [INR] ≤1.5 or thrombocytes ≥50,000/μL). We performed TACE in patients with intermediate stage HCC to downsize and reduce the number of tumors before thermal ablation in both groups. We excluded patients with (1) extrahepatic metastases, (2) vascular invasion, and (3) decompensated liver function. The characteristics of patients and tumors are shown in Table 1.

### 2.3. Thermal Ablation Protocol

Informed consent forms were obtained from all patients in both groups. All ablations were performed under analgosedation and as an outpatient procedure in both groups by two consultants in interventional radiology with an experience of more than 10 years. The patients received intravenously pre-interventional antibiotic prophylaxis. Current blood count as well as coagulation status (Thrombocytes count, INR and PTT) were required. The final abdominal contrast-enhanced magnetic resonance imaging (CEMRI) or contrast-enhanced computed tomography (CECT) scan of the patients was studied and evaluated closely, and a planning CT-scan was carried out to assess the puncture angle directly before the ablation. After thoroughly disinfecting the skin and covering it with a sterile drape, the local anesthetic was injected. Electrocardiography and vital parameters such as pulse, blood pressure, respiratory rate, and oxygen saturation were monitored during the treatment. When a complete ablation was reached, the active microwave antenna or laser applicator was carefully removed, and the needle track was sealed. The patients were placed on bed rest for 4 to 6 h following the treatment and were continuously monitored. If necessary, pain medications were administered to the patients who complained of post-interventional pain.

### 2.4. MWA Procedure

We used the Emprint™ with Thermosphere™ Technology Covidien system in the treatment of our HCC patients. After local anesthesia, a small skin incision was made to percutaneously insert the microwave antenna into the target lesion. Following the insertion and positioning of the antenna in the lesion under CT guidance (Somatom Sensation 64; Siemens), the thermal ablation was performed according to the manufactural protocol. For monitoring the ablation process, CT fluoroscopic scans were repeatedly performed.

### 2.5. LITT Procedure

The puncture of the tumor and insertion of the laser applicator was performed under CT guidance (Multislice-CT Somatom Plus 4 Volume Zoom, Siemens). Depending on the size of the lesion, up to 4 laser applicators were simultaneously inserted. We also used the pull-back technique in order to ensure complete ablation of the tumor. After the insertion of the laser applicator, the patient was transferred to another room, where the lesion was ablated using the Nd-YAG laser at a wavelength of 1064 nm under MR-guidance (Avanto, Siemens). T1-weighted imaging was used for ablation and its live monitoring. We performed the LITT using the laser application kit SOMATEX, which is compatible with MRI. This laser-system is cooled with normal saline and heat resistant up to 400 °C.

### 2.6. Follow-Up Protocol

Follow-up imaging was done using CEMRI. The contrast agent was injected intravenously with a flow rate of 1–2 mL/s and rinsed with normal saline. To evaluate the ablation area and detect possible late-occurring complications, the first CEMRI scan took place 24 h post-intervention. The patients received 4 follow-up visits 3-months apart within the first year and 2 follow-up visits bi-annually thereafter.

### 2.7. Data and Statistical Analysis

All cases were evaluated according to the number and location of tumors, axial diameter of tumor and ablation area, duration of ablation, technical success, complete ablation, local tumor progression (LTP), intrahepatic distant recurrence (IDR), overall survival (OS) time, disease-free survival (DFS) time, and complications. Since the included patients were similar and without significant differences according to gender, age, size, or number of tumors between both groups, a propensity score matching was not required.

LTP was defined as developing a new HCC lesion directly adjacent to the ablation area and/or an increase in the size of the ablation area at the follow-up. IDR was defined as developing new HCC lesions in non-ablated liver segments away from the originally treated tumor. The OS rates were calculated starting at the date of diagnosis or at the date of the first treatment until the date of the last follow-up or death. The DFS was calculated starting at the date of the treatment until the date of LTP or IDR or death. The IDR- and LTP-free survival rates were calculated starting at the date of the treatment until the date of the first event. We divided the complications, according to the guidelines of the Society of Interventional Radiology [8], into two groups: (1) major complications that required further interventional and/or surgical treatments, in which the patients needed to be hospitalized for a longer time and (2) minor complications that did not require any further treatment, such as mild hemorrhage and asymptomatic pleural or pericardial effusion. Technical success was defined as a correct positioning of the antenna or applicator, according to the protocol in the tumor under image-guidance, without any disruption of the treatment due to technical reasons. Initial complete ablation was defined as full coverage of the treated tumor 24h post-ablation at the CEMRI after the first ablation.

Statistical analysis of this study was done using SPSS^®^ (Statistical Package for the Social Sciences, GradPack 27.0 Premium for Mac). The OS and DFS rates were calculated by the Kaplan-Meier method and the IDR- and LTP-free survival rates were analyzed using competing risk analysis. The log-rank test was used for the comparison of the survival rates. We used the Chi-square test and Fisher’s exact test to compare the categorical variables. The Mann-Whitney-U test was used to compare the samples since the values were not normally distributed. A *p*-value of ≤ 0.05 was considered statistically significant.

## 3. Results

The results are summarized in Table 2.

### 3.1. Indices of Tumors and Ablation Area

The mean axial tumor diameter was 2.2 ± 0.92 cm in the MWA-group and 2.4 ± 0.94 cm in the LITT-group. The mean axial diameter of the ablation area was 4.4 ± 1 cm in the MWA-group and 5.3 ± 1.8 cm in the LITT-group (*p*-value: 0.0001). The difference in the size of the ablation area was significant.

### 3.2. Duration of Ablation

The mean ablation time was 10.5 ± 5.3 min in the MWA-group and 16.7 ± 7.4 min in the LITT-group. The difference in the ablation time was significant (*p*-value < 0.001).

### 3.3. Technical Success and Complete Ablation Rate

The complete ablation rate was 97.7% (425/435) in the MWA-group and 98.7% (74/75) in the LITT-group. The difference in the complete ablation rate was not significant (*p*-value > 0.99).

Ten MWA sessions and one LITT session were additionally performed in order to reach a complete ablation in the residual lesions.

### 3.4. Therapy Response

LTP was reported in 6% (15/250) of the patients in the MWA-group and in 3.8% (2/53) of the patients in the LITT-group. A total of 46% (115/250) of the patients in the MWA-group and 64.2% (34/53) in the LITT-group developed IDR.

**Figure 1:**
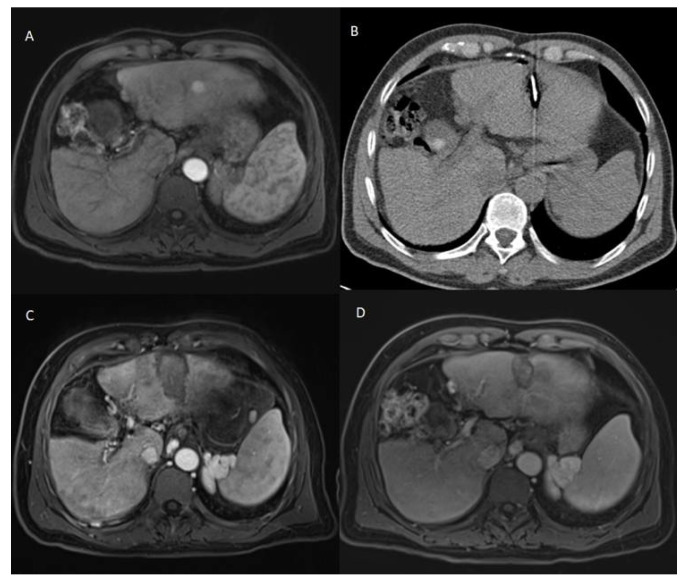
60 years old male patient with chronic hepatitis B virus, mild liver cirrhosis, and HCC lesion in the left liver lobe, who was treated with MWA. (**A**) Pre-treatment CEMRI showed a hyperenhancement of the lesion in the arterial phase. (**B**) During MWA. (**C**) 24h post-ablation CEMRI showed the completely ablated lesion. (**D**) 2-years post-ablation CEMRI showed a complete remission after MWA. The survival time of this patient was 21 months starting from the date of ablation until last contact.

**Figure 2:**
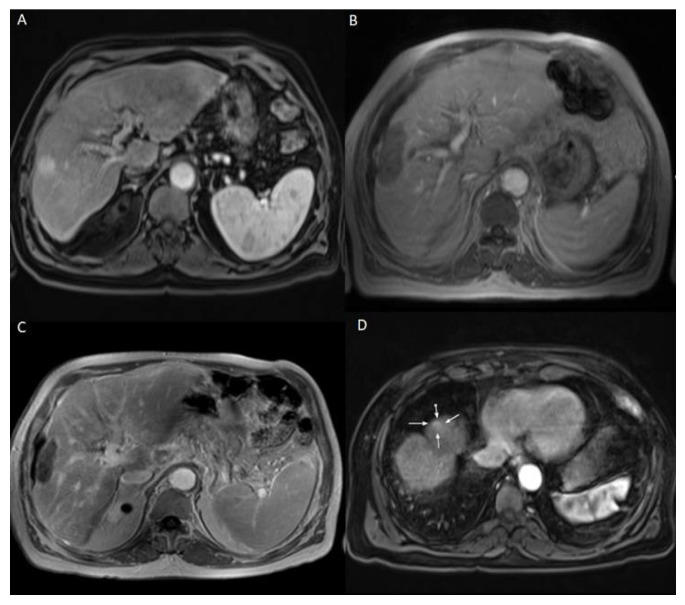
69 years old male patient with chronic hepatitis C virus, liver cirrhosis, and HCC lesion in the right liver lobe, who was treated with LITT. (**A**) Pre-treatment CEMRI showed an arterial hyperenhancement of the lesion. (**B**) 24 h post-ablation CEMRI showed a large post-ablation zone, and the lesion was fully ablated. (**C**,**D**) At the 3-months post-ablation CEMRI, the size of the ablation zone was getting decreased without LTP, but there was a hyperenhancement in untreated liver segment (white arrows), which was correlated with IDR. TACE was performed to treat the recurrent HCC and the patient lived for 33 months after LITT until death.

The patients who developed LTP or IDR were treated by MWA, LITT, or TACE, depending on the number, size, and location of the new HCC lesions. The decision was taken by the multidisciplinary tumor board. Examples of Patients’ cases are shown in Figure 1 and Figure 2.

### 3.5. Survival Rates

In the MWA-group, the mean follow-up time was 31.5 ± 22 months. The mean follow-up time was 35.7 ± 26 months in the LITT-group. The difference in the follow-up time between both groups was not significant (*p*-value: 0.378).

**Figure 3:**
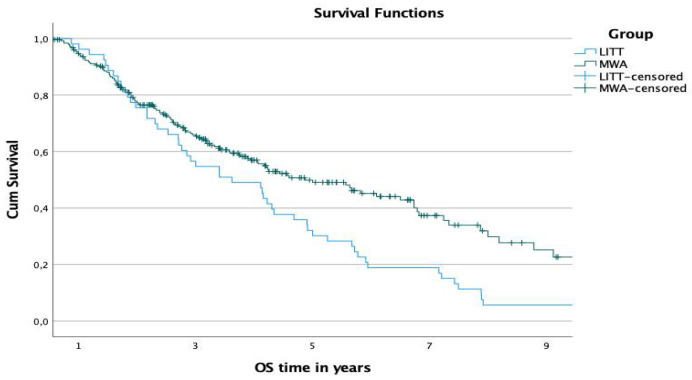
Comparison of OS rates of both groups starting at the date of diagnosis.

The 1-, 3-, and 5-year OS rates starting at the date of diagnosis were 94.3%, 65.4%, and 49.1% in the MWA-group and 96.2%, 54.7%, and 30.2% in the LITT-group, respectively (Figure 3). The difference in the survival time was significant (*p*-value: 0.002).

**Figure 4:**
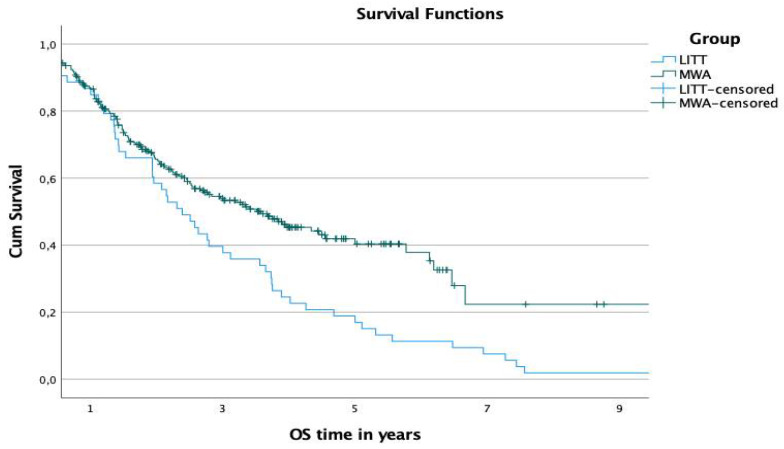
Comparison of OS rates of both groups starting at the date of ablation.

The 1-, 3-, and 5-year OS rates from the ablation date were 86.6%, 53.4%, and 40.4% in the MWA-group and 85%, 37.7%, and 17% in the LITT-group, respectively (Figure 4). The difference in the overall survival time was significant (*p*-value: 0.001).

**Figure 5:**
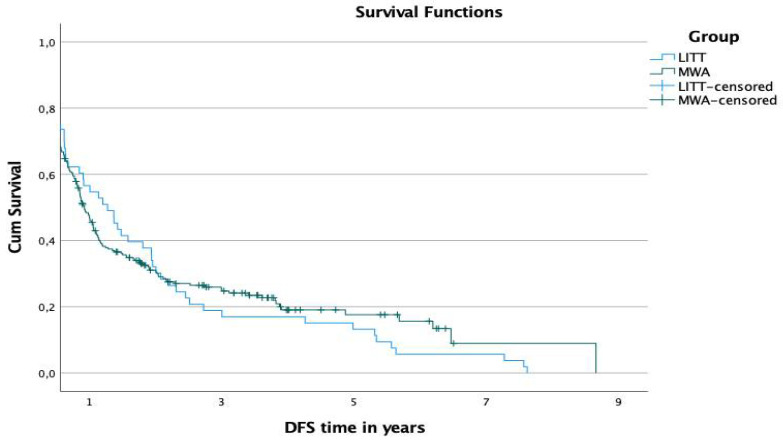
Comparison of DFS rates of both groups.

The 1-, 2-, and 3-year disease-free survival (DFS) rates were 45.9%, 30.6%, and 24.8% in the MWA-group and 54.7%, 30.2%, and 17% in the LITT-group, respectively (Figure 5). The difference in the DFS was not significant (*p*-value: 0.719).

The 1-, 2-, and 3-year LTP-free survival rates were 95.2%, 93.8%, and 93.8% in the MWA-group and 96.2%, 96.2%, and 96.2% in the LITT-group, respectively. The differences in the LTP-free survival were not significant (*p*-values: 0.67, 0.43, and 0.43, respectively).

The 1-, 2-, and 3-year IDR-free survival rates were 55.6%, 46.4%, and 42.2% in the MWA-group and 64.2%, 49%, and 39.6% in the LITT-group, respectively. The differences in the IDR-free survival were not significant (*p*-values: 0.27, 0.49, and 0.85, respectively).

### 3.6. Complications

There were no procedure-related deaths reported in both groups.

The overall complication rate was 2.9% (13/445) in the MWA-group and 7.9% (6/76) in the LITT-group. The difference in the overall rates of complications was significant (*p*-value: 0.045). There were no major complications reported in the LITT-group and there was only one case of major complications in the MWA-group at a rate of 0.2% (1/445), where a large post-interventional hemorrhagic pleural effusion was treated with a thoracic drainage. The difference in the rate of major complications was not significant (*p*-value > 0.99). The rate of minor complications was 2.7% (12/445) in the MWA-group and 7.9% (6/76) in the LITT-group. The difference in the rate of minor complications was significant (*p*-value: 0.034). The complications are summarized in Table 3.

## 4. Discussion

Image-guided interventional treatments in general and the percutaneous thermal ablation are gaining an increasingly important role in the therapy of HCC. The decision whether HCC should be treated by surgery, image-guided thermal ablation, intra-arterial methods, or by radiation therapy must be taken by interdisciplinary teams consisting of surgeons, interventional radiologists, oncologists, hepatologists, and radiation oncologists, and must take into consideration the location and size of the tumor, liver function, existence of extrahepatic manifestation, and overall health of the patients and their preference.

The main challenge in treating HCC patients by local ablative techniques such as MWA or LITT is not only the development of LTP, but also the development of IDR. Here, we show that 46% of the patients in the MWA-group and 64.2% in the LITT-group developed IDR, but only 6% in the MWA-group and 3.8% in the LITT-group developed LTP. Other authors reported rates of LTP in ranges of 8.8–29.2% [9,10,11,12,13,14,15,16] and in ranges of 2–19.5% [17,18,19] while evaluating MWA and LITT of HCC, respectively.

The rates of LTP and complications were low in both groups, which demonstrates that LITT and MWA are safe and effective in the local control of HCC.

In other studies which investigated MWA and LITT, the rates of major complications were in ranges of 1–3.8% [9,11,13,14,15,16,20,21] and 0.0–1.5% [17,18,22,23,24], respectively. In our LITT-group, there were no major complications reported, and there was only one case of major complication at a rate of 0.2% in the MWA-group.

Even though the mean diameter of HCC was larger in the LITT-group than in the MWA-group, the LTP rate was lower in the LITT-group than in the MWA-group. The reason behind the lower LTP rate and better local tumor control in the LITT-group can be attributed to the larger ablation areas, and thus the larger ablative margins than in the MWA-group. We achieved an initial complete ablation in 97.7% of the tumors in the MWA-group and in 98.7% of the tumors in the LITT-group. Prior studies which examined MWA and LITT reported initial complete ablation rates of 71.1–98.5% [9,20,25] and 66.7–98% [17,23], respectively.

We found that HCC patients treated by MWA had significantly longer OS time than patients treated by LITT. The DFS time was similar in both groups without any significant difference. The differences between the groups are even higher for a matched data analysis (results not shown). In previous studies that analyzed MWA of HCC, the 1- and 5-year OS rates were in the range of 82.7–98.4% [9,10,11,12,13,20,21,25,26,27] and 21–61.3% [10,11,13,26,27], respectively, and the 3-year DFS rate was in the range of 30.6–41.8% [9,13,26,27]. In addition, studies that have investigated LITT of HCC showed a 1-year OS rate in the range of 88.6–99% [17,18,19,22,28] and a 3-year OS rate in the range of 54–68% [17,18,19,22,29]. These survival rates are comparable to our rates in both groups. We reported 1- and 5-year OS rates of 94.3% and 49.1%, respectively, starting at the date of diagnosis, and a 3-year DFS rate of 24.8% in the MWA-group. In our LITT-group, the 1- and 3-year OS rates were 96.2% and 54.7%, respectively, starting at the date of diagnosis.

It is also important to highlight that the development of LTP and occurrence of complications in HCC patients treated by local ablative treatments does not only depend on the technique of the thermal ablation, but also on the size and location of the tumor, since some tumors are located in challenging positions adjacent to the gallbladder, large vessels, or diaphragm, for example [30].

The main benefit of LITT over MWA is the live monitoring of the procedure under MRI guidance. This allows delivery of the accurate amount of energy needed to ablate the lesion, which in turn saves the normal hepatic tissue from unnecessary destruction during ablation [31]. On the other hand, this method increases the likelihood of complete ablation.

Our study has some limitations. Firstly, it was a retrospective study, where some important parameters for a well-matched study were not available, and we believe a prospective randomized study would investigate and evaluate the efficacy and safety of MWA and LITT in the treatment of HCC more accurately. Secondly, this study did not consider the possible effect of pretreatments, such as TACE. Lastly, we believe a multicenter study may include a larger population of patients and combine expertise from different institutes, which would evaluate this approach more comprehensibly.

## 5. Conclusions

Here, we present studies that show MWA and LITT as minimally invasive local techniques that are both effective and safe options for the treatments of patients with HCC. MWA should be favored over LITT for the treatment of HCC, since the patients in the MWA-group had significantly longer overall survival time than the patients in the LITT-group.

## Figures and Tables

**Table 1:** Characteristics of patients and tumors.

Parameter	MWA	LITT	*p*-Value
Number of patients	250	53	
Mean age	66 ± 10 yrs.	67.5 ± 8 yrs.	0.432
Gender (W:M)	52:198	12:41	0.91
Number of tumors	435	75	
Mean diameter of tumor	2.2 ± 0.92 cm	2.4 ± 0.94 cm	
Patients with solitary tumor	56% (140/250)	68% (36/53)	0.148
Patients with two tumors or more	44% (110/250)	32% (17/53)	
Tumors ≤2 cm	53.8% (234/435)	41.3% (31/75)	0.061
Tumors >2 cm	46.2% (201/435)	58.7% (44/75)	
Caudate lobe	1.6% (7/435)	0% (0/75)	
Right lobe	64% (278/435)	72% (54/75)	
Left lobe	31% (135/435)	26.7% (20/75)	
Bilobar	3.4% (15/435)	1.3% (1/75)	

**Table 2:** Results and outcome.

Parameter	MWA	LITT	*p*-Value
Mean diameter of ablation area	4.4 ± 1 cm	5.3 ± 1.8 cm	0.0001
Number of sessions	445	76	
Technical success	100% (445/445)	100% (76/76)	
Initial complete ablation	97.7% (425/435)	98.7% (74/75)	>0.99
Mean ablation time	10.5 ± 5.3 min	16.7 ± 7.4 min	<0.001
LTP	6% (15/250)	3.8% (2/53)	
IDR	46% (115/250)	64.2% (34/53)	

**Table 3:** Complications.

Parameter	MWA	LITT	*p*-Value
Overall Complication rate	2.9% (13/445)	7.9% (6/76)	0.045
Major complications	0.2% (1/445)	0.0%	>0.99
Minor complications	2.7% (12/445)	7.9% (6/76)	0.034
Mild hemorrhage	1.35% (6/445)	2.6% (2/76)	
Pleural effusion	1.35% (6/445)	5.3% (4/76)	
Pericardial effusion	0.2% (1/445)	0.0%	

## Data Availability

Data is available upon request.
